# Fabrication and Bonding Strength of Sn-Decorated MWCNT-Reinforced Sn-3.0Ag-0.5Cu Composite Solder Joints: Reflow vs. IPL Soldering

**DOI:** 10.3390/ma19112188

**Published:** 2026-05-22

**Authors:** DongGil Kang, HoGyeong Seong, JaeJun Yoon, MinJae Sung, JinHo Joo, JeongWon Yoon, SeungBoo Jung

**Affiliations:** 1 School of Advanced Materials Science & Engineering, Sungkyunkwan University, 2066 Seobu-ro, Jangan-gu, Suwon 16419, Republic of Korea; 2 Department of Semiconductor Convergence Engineering, Sungkyunkwan University, 2066 Seobu-ro, Jangan-gu, Suwon 16419, Republic of Korea; 3 School of Advanced Materials Science & Engineering, Chungbuk University, Chungdae-ro, Seowon-gu, Cheongju-si 28644, Republic of Korea; jwyoon@chungbuk.ac.kr

**Keywords:** Sn-3.0Ag-0.5Cu, Sn nanoparticles, MWCNT, intense pulsed light, intermetallic compounds

## Abstract

The rapid advancement of microelectronic packaging has created a critical need for lead-free solder joints with enhanced mechanical and thermal reliability. This study introduces a novel approach to improve Sn-3.0Ag-0.5Cu (SAC 305) solder joints by incorporating Sn-decorated multiwalled carbon nanotubes (MWCNTs). To address the poor wettability and agglomeration of carbon nanotubes in molten solder, MWCNTs were functionalized and uniformly coated with ~70 nm Sn nanoparticles via electroless plating. Soldering was conducted using intense pulsed light (IPL), a rapid, energy-efficient heat source, and was compared with conventional reflow soldering. The study systematically investigated the influence of MWCNT content (0, 0.05, 0.1, and 0.2 wt.%) and IPL soldering conditions with pulse numbers: 27–36 for shear tests, and 30–42 for drop impact tests. IPL processing produced thinner Cu_6_Sn_5_ IMC layers than reflow soldering due to its shorter duration. The composite solder with 0.1 wt.% Sn-decorated MWCNTs achieved the highest density, superior thermal dissipation in LED packages, and maximum shear strength and drop impact resistance. These results demonstrate that optimizing Sn-MWCNT content, especially at 0.1 wt.%, and precisely controlling IPL energy can yield highly reliable, mechanically robust, and thermally efficient lead-free solder joints for advanced electronic packaging.

## 1. Introduction

Ongoing trends toward miniaturization, multifunctionality, and high-speed operation in electronic devices have pushed packaging technologies to their limits. In electronic assemblies, solder joints function as both mechanical connectors and critical pathways for electrical signal transmission and heat dissipation. After the global ban on lead-containing solders due to environmental and health concerns, Sn-Ag-Cu (SAC) alloys—especially SAC 305 (Sn-3.0 wt.%Ag-0.5 wt.%Cu)—emerged as the industry standard for lead-free soldering, owing to their relatively low melting temperature, process compatibility, and satisfactory mechanical properties [1,2,3,4].

As electronic components shrink and power densities rise, the reliability of conventional SAC 305 solder joints has become a significant concern. Under harsh service conditions—such as vibration and mechanical shock in automotive infotainment modules—solder joints often fail, with the solder/Cu substrate interface being the most vulnerable region. During soldering and subsequent operation, interdiffusion of Cu, Sn, and Ag results in the formation of intermetallic compounds (IMCs) such as Cu_6_Sn_5_, Cu_3_Sn, and Ag_3_Sn. While a thin IMC layer is essential for metallurgical bonding, excessive IMC growth and the formation of brittle Cu_3_Sn can initiate and propagate cracks, greatly reducing resistance to impact and drop loading [5,6,7,8].

To improve solder joint reliability, nanomaterial-reinforced composite solders have gained significant attention. Potential reinforcements include carbon-based nanomaterials (graphene, graphite, diamond); ceramic particles (Al_2_O_3_, Si_3_N_4_, BN, ZrO_2_, TiB_2_, AlN); and high-melting-point elements, such as Mo and rare-earth additives [9,10,11,12,13,14,15,16,17]. Multi-walled carbon nanotubes (MWCNTs) are especially attractive due to their outstanding mechanical strength, high aspect ratio, thermal conductivity above 3.5 kW/m·K [18,19], and ability to form a percolated network in the solder matrix. Uniformly dispersed MWCNTs in the SAC 305 matrix can hinder dislocation motion, suppress excessive IMC growth, and enhance joint toughness.

However, practical implementation of MWCNTs in composite solders is hindered by poor wettability and severe agglomeration. Due to chemical incompatibility between the carbon network and molten Sn, pristine MWCNTs exhibit poor wetting with liquid solder, leading to clustering or flotation during processing. These agglomerates act as stress concentrators and defects, which degrade mechanical performance. To address interfacial incompatibility, previous studies have decorated MWCNTs with metals such as Cu, Ni, and Ag. In this work, we developed a chemical process to decorate MWCNT surfaces with tin (Sn) nanoparticles, improving compatibility with the SAC 305 matrix and promoting uniform dispersion in the solder joint. During soldering, the Sn coating melts or diffuses into the SAC 305 matrix, allowing the carbon nanotubes to anchor and distribute uniformly. In addition to material optimization, the soldering process should also aim to reduce energy consumption. Conventional reflow soldering heats the entire PCB assembly by convection at elevated temperatures for at least 300 s, increasing energy use and risking damage to temperature-sensitive components. To overcome these drawbacks, we used IPL as the heat source. IPL is a rapid thermal processing technique that delivers high-energy light pulses from a xenon lamp within milliseconds. By selectively heating solder paste and dark surfaces via photothermal absorption, IPL enables localized melting while minimizing overheating of surrounding materials.

This study systematically investigated the synthesis of Sn-decorated MWCNTs and their incorporation into SAC 305 composite solders (hereafter called the composite solder) processed by IPL. We evaluated the effects of MWCNT content and IPL energy on interfacial reactions, shear strength, and drop-impact reliability of solder joints. Results were compared with those from conventional reflow soldering to identify optimal processing conditions for high-reliability electronic applications.

## 2. Materials and Methods

**Figure 1:**
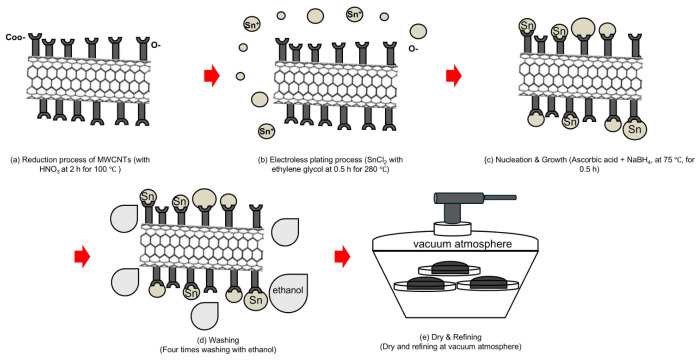
Schematic diagram of the synthesis process for Sn-decorated multi-walled carbon nanotubes (MWCNTs).

**Figure 2:**
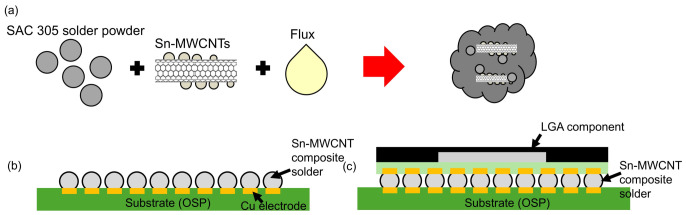
Fabrication steps of (**a**) SAC 305 composite solder paste, (**b**) composite solder ball for shear test, and (**c**) board-level package for drop impact test.

Figure 1 presents a schematic of the synthesis process for Sn-decorated multiwalled carbon nanotube (Sn-MWCNT) particles. Pristine MWCNTs (Bioneer Corp., Daejeon, Republic of Korea; outer diameter: 20–30 nm, length: 10–30 μm) were functionalized by treatment with HNO_3_ at 100 °C for 2 h, introducing carboxyl and hydroxyl groups (–COOH and –OH). The functionalized MWCNTs were then dispersed in ethylene glycol and a 0.1 M SnCl_2_ solution for the polyol synthesis. This mixture was heated to 280 °C for 30 min. Subsequently, the solution was treated with ascorbic acid and NaBH_4_ at 75 °C for 30 min to promote the nucleation and growth of Sn nanoparticles. The resulting product was washed multiple times with ethanol and vacuum-dried at 25 °C for 12 h [3,4]. The uniform distribution of spherical Sn nanoparticles (~70 nm in diameter) on MWCNT surfaces was observed by TEM (JEM-F200, Jeol, Tokyo, Japan). SAC 305 solder powder (20–35 µm particle size) was combined with Sn-MWCNTs (0, 0.05, 0.1, 0.2 wt.%) and commercial flux (Alpha EF9301, Alpha metals, Siheung, Republic of Korea) at 11.8 wt.% and were ultra-sonicated at 25°C for 20 min. And then the Sn-MWCNT composite solder using a planetary the Sn-MWCNT a planetary paste mixer (ARE-310, Thinky Co., Tokyo, Japan) for 1.5 min of mixing and 1 min of deforming to produce homogeneous composite solder pastes, as shown in Figure 2a. The chemical composition, flux, and melting point of the composite solder paste used in this study are listed in Table 1. Specimen specifications for shear test and drop impact tests are summarized in Table 2.

Composite solder pastes were screen-printed onto OSP-finished FR-4 substrates (Kowon Circuit Co., Ltd., Ansan, Republic of Korea) using 150 µm thick stainless steel stencils. For shear strength evaluation, solder balls (Ø 240 µm) were formed via either IPL soldering (IPL station, PSTEK Co., Gunpo-si, Republic of Korea) or conventional reflow soldering (RF-430-N2, Japan Pulse Laboratory Co., Ltd., Isesaki-shi, Japan). For drop impact reliability tests, LGA components (A-CABGA196, Amkor Tech., Tempe, AZ, USA; 15 × 15 × 1.5 mm^3^, 196 I/Os) were mounted on daisy-chain FR-4 test boards (77 × 132 × 1.0 mm^3^).

Both soldering processes were conducted at a peak temperature of 250 °C. Reflow soldering required a total duration of 300 s, while IPL soldering used a Xenon flash lamp system (Xenon lamp, PSTEK Co., Gunpo-si, Republic of Korea) with a 2.75 pulse width, and pulse numbers were 27, 30, 33, and 36 pulses (shear specimens) for shear test. The pulse width of drop impact test was 3.25 ms, and pulse numbers were 30, 36, and 42 pulses for drop specimens. The frequencies for both shear and drop impact tests were 3 Hz, with total processing times of 9–14 s, as detailed in Table 3.

Interfacial microstructures and intermetallic compound (IMC) layers were analyzed by FE-SEM (SEM 3300, Ciqtek, Hefei, China). The shear strength of composite solder joints on 200 µm diameter pads was assessed following the JEDEC JESD22-B117 standard [20], using maximum shear force and total fracture energy to failure. Drop impact reliability of LGA packages made with the four composite solders by IPL or reflow soldering was evaluated on a drop test machine (SD-10, L.A.B Equipment Inc., Itasca, IL, USA) according to JESD22-B110A [21] and JESD22-B111 standards [22]. Failure was defined as the number of drops until resistance increased by more than 20% from the initial value.

Composite solder density was measured using the Archimedes method Thermal performance was assessed indirectly by measuring LED package surface temperatures with infrared thermography after 30 min of steady-state operation at the rated current.

## 3. Results

### 3.1. Synthesis of Sn-Decorated MWCNTs and Microstructural Characteristics

**Figure 3:**
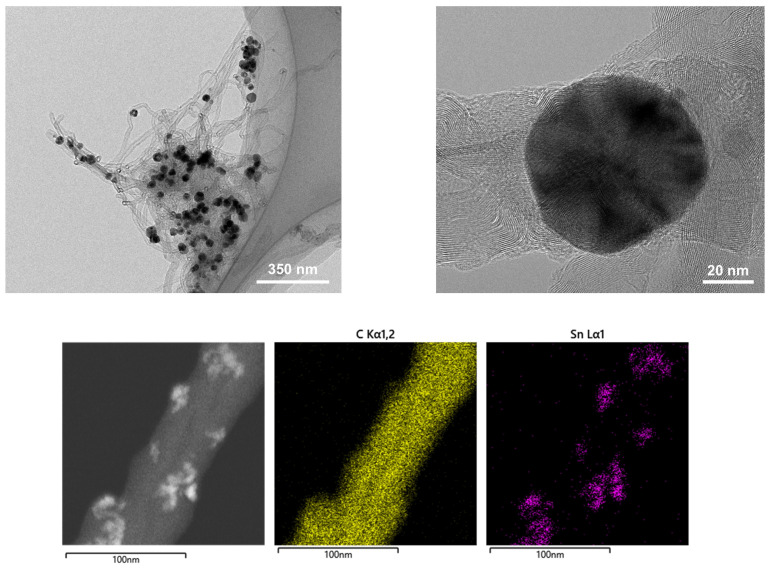
TEM images of Sn-decorated MWCNT particle (70 nm) and EDS analysis result.

The microstructural integrity of composite solders depends strongly on the quality of the reinforcement material. MWCNTs added to the solder matrix inherently provide high thermal conductivity (up to 3.5 kW/m·K), but this can drop by up to an order of magnitude due to alignment and agglomeration within the solder. Thus, achieving uniform dispersion is essential. TEM images, and EDS analysis result (Figure 3 and Table 4) show that the synthesized Sn-MWCNTs display highly uniform Sn nanoparticle decoration. During electroless plating, functional groups on the MWCNT surface serve as nucleation sites for Sn ions, enabling consistent and controlled deposition. The small Sn nanoparticle size (~70 nm) ensures gradual melting during soldering and good blending within the SAC 305 matrix, improving wettability and enhancing physical bonding through network formation. In contrast, untreated MWCNTs tend to agglomerate and may be pushed to grain boundaries or the solder surface during solidification.

### 3.2. Densities, Thermal Emission, and Microstructures of Composite Solder After IPL Soldering and Reflow Soldering

**Figure 4:**
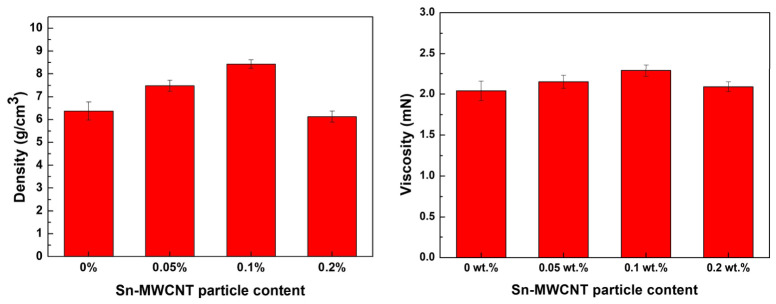
Densities and viscosity of composite solder with various contents of MWCNT particle.

Four composite solder pastes were subjected to conventional reflow soldering, and their densities were measured using the Archimedes principle. The measured densities reflect microstructural integrity and potential defect formation influenced by Sn-MWCNT addition. Figure 4 shows the variation in composite solder density as a function of Sn-decorated MWCNT content. The density of composite solders increased up to 0.1 wt.% (8.43 g/cm^3^) Sn-MWCNT and decreased at higher contents, such as 0.2 wt.% (6.13 g/cm^3^). This variation is closely related to reduced wettability between the solder matrix and Sn-decorated MWCNTs, as well as possible MWCNT agglomeration. The observed decrease in density beyond 0.1 wt.% Sn-MWCNT indicates increased void formation in the composite solder, which may reduce thermal conductivity due to enhanced phonon scattering at void interfaces and MWCNT clusters. The wettability was measured using a wetting balance tester (SWB-2, Malcom, Tokyo, Japan). It was observed that the viscosity increased as the Sn-MWCNT particle content increased, and the highest value of 2.29 mN was obtained at 0.1 wt.%.

**Figure 5:**
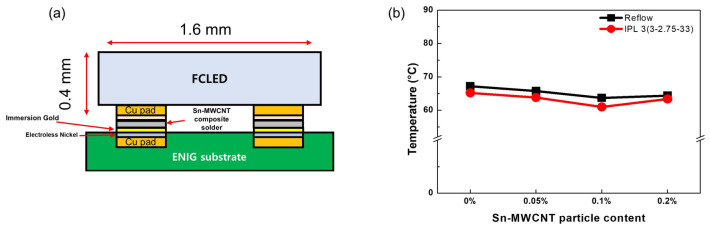
Thermal emission of LED package assembled with composite solder: (**a**) schematic diagram of LED package, and (**b**) surface temperature.

Figure 5 shows the thermal emission profiles of LED packages assembled with various IPL-composed composite solders using reflow soldering. The thermal dissipation performance of composite solders was indirectly evaluated from these LED packages. As shown in Figure 5, the LED package assembled with 0.1 wt.% Sn-MWCNT-reinforced composite solder via IPL soldering exhibited the lowest surface temperature. This result is attributed to the high density of the 0.1 wt.% Sn-MWCNT composite solder (Figure 4), which correlates with enhanced thermal conductivity. Superior thermal conductivity facilitates efficient heat transfer from the LED die to the PCB substrate, minimizing the LED device’s temperature when assembled with 0.1 wt.% Sn-MWCNT composite solder [23,24].

**Figure 6:**
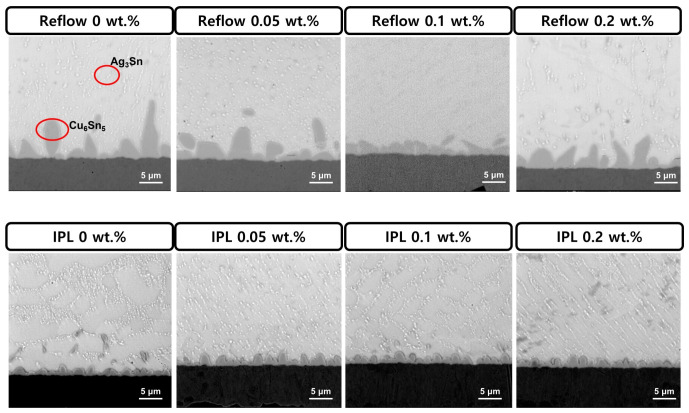
Cross-sectional micrographs of composite solder joint of reflow, and IPL condition 3 (3 Hz-2.75 ms-33).

Figure 6 displays cross-sectional microstructures of composite solder/Cu joints fabricated by IPL and reflow soldering, confirming Cu_6_Sn_5_ intermetallic compound (IMC) formation in all cases. Microstructural analysis of SAC 305 composite solder joints after IPL soldering revealed a more wavelike Cu_6_Sn_5_ intermetallic compound (IMC) layer at the interface between the composite solder and the OSP-finished Cu pad compared to reflow soldering. IPL joints consistently exhibited thinner IMC layers compared to reflow joints, due to the much shorter processing time (~1/25th), which suppresses atomic interdiffusion. Previous studies have shown that, in conventional SAC 305 solder joints, a Cu_6_Sn_5_ IMC forms predominantly at the OSP-finished Cu interface, with only a very thin Cu_3_Sn IMC layer also present [25,26]. In contrast, in the current composite solder joints, the addition of MWCNTs suppresses the growth of intermetallic phases, with only Cu_6_Sn_5_ detected at the interface. Fine, needle-like or particle-shaped Ag_3_Sn compounds are widely dispersed within the bulk composite solder matrix. In composite solders with Sn-MWCNTs, the interfacial IMC layer is thinner than in pure SAC 305 joints. The functionalized carbon nanotubes serve as physical barriers during the liquid-state reaction and cooling, restricting the rapid diffusion of Cu and Sn atoms and suppressing the excessive growth of scallop-shaped Cu_6_Sn_5_ crystals [12,13,14,15,16,17,18,19,27].

**Figure 7:**
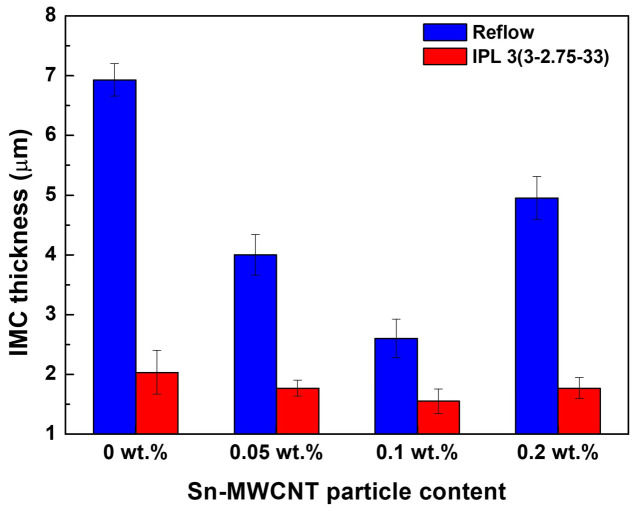
Thickness of IMC formed in composite solder joints.

Figure 7 quantifies IMC thickness as a function of soldering method and Sn-decorated MWCNT content, showing a decrease up to 0.1 wt.% MWCNT (from 6.9 to 2.6 µm for reflow soldering, from 2.0 to 1.5 µm for IPL soldering) as the particles physically block Sn-Cu diffusion; IPL soldering produced significantly thinner IMCs than reflow. In reflow soldering (at 250 °C for 300 s), adequate processing time allows metal diffusion, suppressing IMC growth up to 0.1 wt.% Sn-MWCNT; beyond this, void formation and MWCNT agglomeration reduce the effect. Thinner IMCs formed by IPL and Sn-MWCNT reinforcement enable precise interfacial control, improving joint reliability in advanced packaging. While reflow risks IMC overgrowth and grain coarsening across multiple cycles, which degrade strength, IPL mitigates these issues through rapid processing. However, optimization of IPL pulse count, width, and frequency is essential and depends on component size and PCB substrate mounting density [11,12,13,14,15,16,17,18,19,27,28,29,30].

### 3.3. Shear Strength and Fracture Energy of Composite Solder Joints

**Figure 8:**
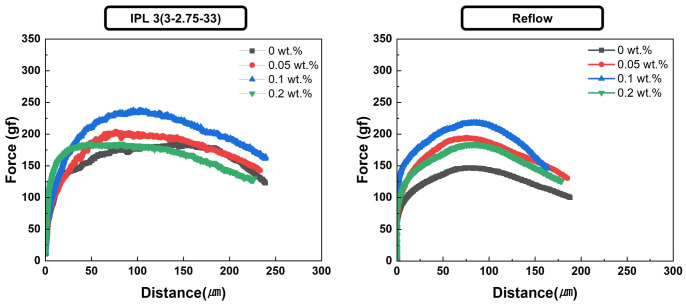
Typical F-X curve of composite solder joints.

This study comparatively assessed the effects of Sn-MWCNT addition on the shear strength of composite solder joints. Shear strength testing by the JESD22-B117 standard is the most common method for evaluating a solder joint’s resistance to static mechanical loads. Four kinds of composite solders were screen-printed onto OSP finished FR-4 substrates, followed by IPL or reflow soldering to fabricate shear test specimens. Figure 8 presents representative force-displacement (F-X) curves from shear tests of composite solder joints. The joint reinforced with 0.1 wt.% Sn-decorated MWCNTs showed the highest shear load, indicating superior mechanical strength compared to other compositions. This peak performance corresponds to optimal density and minimal IMC thickness at this Sn-MWCNT content (Figure 4 and Figure 7), reflecting improved interfacial bonding and reduced defect formation. In addition, a stable Sn-MWCNT network structure within the solder matrix collectively enhanced interfacial bonding and ductility [23,24,25,26,31,32,33,34,35,36,37].

**Figure 9:**
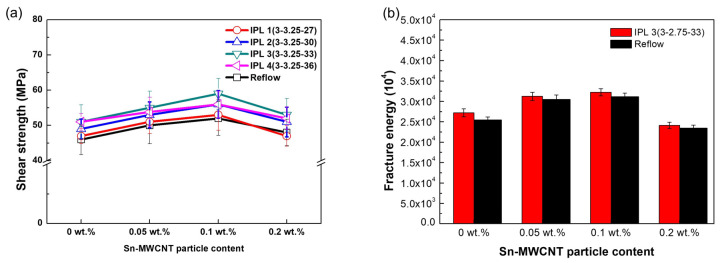
(**a**) Shear strength of composite solder joints with contents of Sn-MWCNT particles, and (**b**) fracture energy.

As shown in Figure 9a, the shear strength of composite solder joints increased with MWCNT content, with the highest shear strength being 59 MPa at 0.1 wt.%, with IPL soldering outperforming 49 MPa with reflow. This improvement results from MWCNT dispersion strengthening, suppression of intermetallic compound growth, and the formation of a network structure in the solder matrix. Increasing IPL pulse count further improved shear strength through enhanced wettability, uniform alloy and Sn-MWCNT distribution, short processing time, and MWCNT-mediated IMC inhibition. As a result, IPL joints achieved shear strengths comparable to or exceeding those of reflow-processed joints. Sn-MWCNTs act as second-phase dispersoids that bridge microcracks and hinder dislocation motion under shear loading, greatly enhancing joint load-bearing capacity and impact resistance. However, with more than 33 IPL pulses, shear strength plateaued or slightly decreased, as excessive IMC layer growth offset the benefits of MWCNT reinforcement [32,33]. Figure 9b presents an analysis of the fracture energy of the composite solder. The fracture energy increases progressively from 0 wt.% up to a content of 0.1 wt.% Sn-MWCNT particle. The maximum fracture energy for both soldering processes is observed at 0.1 wt.%, which recorded values at 32,247 (IPL soldering) and 31,214 (reflow soldering). However, in the case of 0.2 wt.%, the excessive content of Sn-MWCNT particles is attributed to nanoparticle agglomeration in solder matrix, which introduces stress concentrations rather than hindering the crack propagations.

### 3.4. Drop Impact Strength of LGA Package Assembled with Various Composite Solders

The drop impact reliability of LGA packages assembled with composite solders was evaluated by measuring electrical resistance before and after testing. While shear testing assesses static strength, the drop impact test evaluates the solder joint’s ability to withstand sudden dynamic shocks encountered in portable electronics such as smartphones and wearables. Drop reliability was quantified by the number of drops to electrical failure, defined as a resistance increase exceeding 20% of the initial value, under 1500 G conditions according to JEDEC JESD22-B110A and B111. In addition, the drop impact test was conducted with seven specimens for each condition.

**Figure 10:**
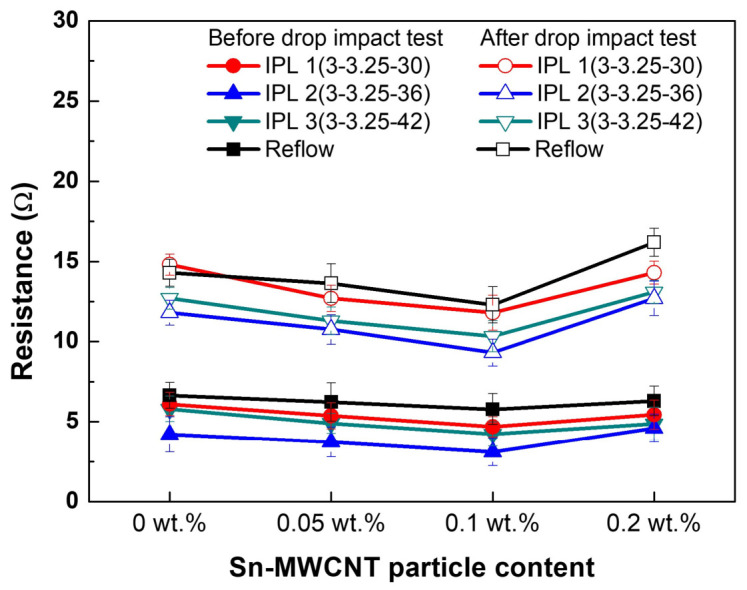
Electrical resistance of LGA component assembled with composite solder before and after drop impact test.

Figure 10 illustrates electrical resistance changes before and after the drop test at 1500 G. LGA packages assembled with 0.1 wt.% Sn-MWCNT-reinforced composite solder exhibited the lowest resistance values, 3.5 before and 9.31 Ω after testing. In contrast, samples with IMC thickness above critical levels or with partial fractures showed significant increases in resistance. The Sn-MWCNTs in the composite solder formed a network structure that acted as conductive bridges, enhancing electrical conductivity and absorbing external stress, leading to a minimal resistance increase at 0.1 wt.% Sn-MWCNT. Furthermore, IPL-soldered specimens exhibited lower overall electrical resistance than reflow-soldered specimens, mainly due to MWCNT-mediated suppression of IMC growth.

**Figure 11:**
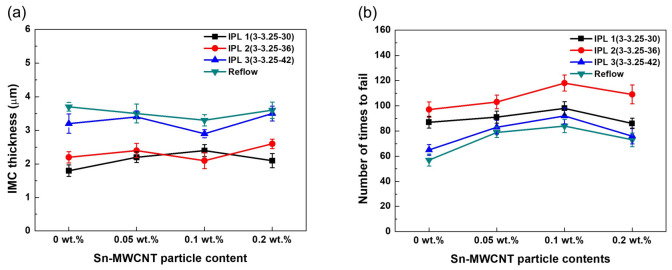
(**a**) Thickness of Cu_6_Sn_5_, and (**b**) drop impact strength of LGA packages with various contents of Sn-MWCNT particle.

As shown in Figure 11a, IMC thickness at LGA package interfaces increased from 1.87 μm to 3.2 μm as IPL pulse counts rose from 30 to 42, yet remained about twice as thin as that produced by conventional reflow soldering. Furthermore, LGA packages produced with 0.1 wt.% Sn-decorated MWCNTs and 36 IPL pulses demonstrated superior drop impact reliability, confirming IPL soldering’s comprehensive superiority over traditional reflow processes under both static mechanical and dynamic impact loading conditions.

Figure 11b shows the drop impact strength of the LGA package with various contents of Sn-MWCNT particles in the composite solder. Thin IMC layers at the solder interface typically promote fracture within the solder bulk, while thick, coarsened IMC layers beyond a critical thickness result in grain boundary fractures within the IMC or along the IMC/coarsened solder interface. Under high-strain-rate impact, cracks rapidly initiate and propagate through the thick IMC or at interfaces with chemically heterogeneous, coarsened solder [25,26,32,33,34,35,36,37,38,39,40].

Overall, composite solders exhibited significantly greater drop impact resistance when processed by IPL soldering compared to reflow soldering. Drop life increased markedly from 57 ± 4.8 cycles (0 wt.% MWCNT) to 84 ± 6.1 cycles (0.1 wt.% Sn-MWCNT) in reflow soldering, and from 97 ± 5.2 cycles (0 wt.% MWCNT) to 118 ± 6.3 cycles (0.1 wt.% Sn-MWCNT) in IPL soldering as reinforcement content increased. LGA components soldered by IPL with 0.1 wt.% Sn-MWCNT showed the highest drop reliability, due to refined solder microstructure, maximized joint density, suppressed IMC growth, and improved energy absorption. However, increasing the MWCNT content from 0.1 to 0.2 wt.% resulted in localized nanotube agglomeration and increased voids, even with Sn decoration, creating stress-concentration sites that reduced impact strength. However, increasing the MWCNT content in the SAC 305 solder matrix from 0.1 to 0.2 wt.% led to localized nanotube bundling and void formation, even with Sn decoration, creating stress concentration sites under high-velocity impact and reducing drop life.

**Figure 12:**
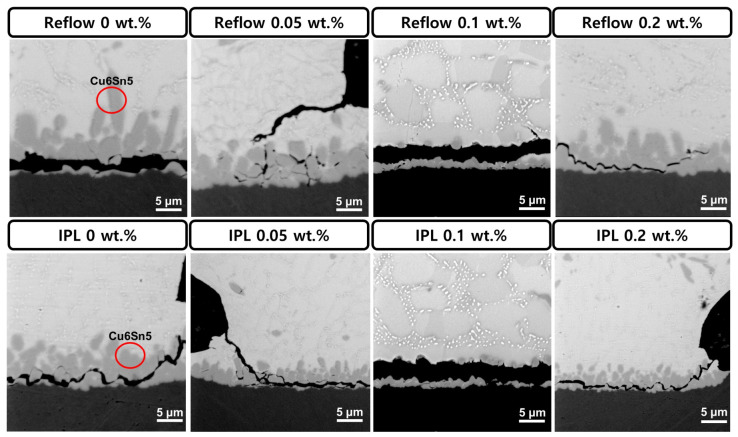
Failure modes and crack propagation in board-level LGA components after drop impact test.

Figure 12 shows the failure modes and crack propagation in board-level LGA components after the drop impact test. The fracture surfaces reveal three distinct failure modes for composite solder joints: (a) due to the interference of crack propagation by Sn-MWCNTs, cracks do not occur only in the IMC layer but propagate through various pathways for Sn-MWCNT contents up to 0.1 wt.%, and (b) predominantly brittle fractures occur within the IMC layer at 0 and 0.2 wt.% Sn-MWCNT.

As detailed above, adding Sn-decorated MWCNTs to SAC 305 solder significantly enhances joint density and thermal dissipation, while also improving shear strength and drop impact resistance. These benefits are most pronounced at the optimal addition level of 0.1 wt.%.

The 0.1 wt.% Sn-MWCNT-reinforced SAC 305 composite solder achieves an optimal balance by suppressing IMC growth, maximizing joint integrity with minimal voids, and forming a percolating nanotube network that effectively disperses stress and absorbs impact energy under high strain-rate conditions. This formulation offers valuable insights for high-reliability electronic packaging applications requiring both static mechanical strength and dynamic drop performance [25,26,31,32,33,34,35,36,37,38,39,40,41,42,43].

## 4. Conclusions

This study systematically examined the preparation and reliability of composite solder joints reinforced with Sn-decorated MWCNTs and fabricated by IPL soldering. Incorporating Sn-MWCNTs effectively suppressed the rapid growth of scallop-shaped Cu_6_Sn_5_ intermetallic compounds at the solder/substrate interface, improving interfacial stability and overall joint performance. The addition of MWCNTs enhanced the electrical conductivity and thermal dissipation of SAC 305 solder, due to the conductive network structure formed by MWCNTs within the composite matrix. IPL soldering also outperformed conventional reflow soldering, as its rapid heating rate and short processing time produced a thinner intermetallic compound layer and a refined solder microstructure. These results demonstrate the highest shear strength of 59 MPa, and C improved the drop impact reliability of 118 cycles for board-level LGA packages. The MWCNTs in the Sn–MWCNT composite solder enhanced mechanical properties through their high tensile strength, their ability to distribute propagated stresses within the solder matrix, and their ability to interrupt fracture. However, insufficient IPL energy resulted in incomplete melting and poor metallurgical bonding. These findings demonstrate that combining Sn-decorated MWCNTs with rapid IPL soldering is a promising strategy for developing green, energy-efficient, and highly reliable electronic packaging materials and processes for future applications.

## Figures and Tables

**Table 1:** Composite solder pastes used in this work.

Type	Solder Powder	SAC 305 Composite	Flux Content	Melting Point
4	20~38 μm	SAC 305 composite solder paste (0, 0.05, 0.1, 0.2% Sn decorated MWCNT particle)	11.8 wt.%	215~220 °C

**Table 2:** Specifications of the test specimens used in this study.

**Shear** **Test**	**Type**	**Opening**	**Pitch**	**Size**	**Ball Size** **(µm)**	**Surface Finish**
	SMD	200 µm	1 mm	1 × 10 × 30 mm^3^	240	OSP
**Drop impact test**	**LGA component** **(Full array)**	**I/O Counts**	**Pitch**	**Size**	**Ball Size** **(µm)**	**Surface Finish**
		196	1.0 mm	1.5 × 15 × 15 mm^3^	500	ENIG
	**Board side** **(SMD)**	196	1.0 mm	1 × 77 × 132 mm^3^		OSP

**Table 3:** Soldering conditions applied in this work.

**Shear Test Condition**						
**Condition**	**Frequency (Hz)**	**Pulse Width (ms)**	**Number** **(n)**	**Temperature** **(°C)**	**Energy (J/cm^2^)**	**Time (s)**
**IPL1**	3	2.75	27	181.8	269.3	9
**IPL2**	3	2.75	30	210.7	299.2	10
**IPL3**	3	2.75	33	215.3	329.1	11
**IPL4**	3	2.75	36	226.6	359	12
**Reflow**	Peak temperature: 250 °C				-	300
**Drop Impact Test Condition**						
**IPL (*n* = 30)**	3	3.25	30	220.4	353.6	10
**IPL (*n* = 36)**	3	3.25	36	236.7	424.3	12
**IPL (*n* = 42)**	3	3.25	42	250.3	494	14
**Reflow**	Peak temperature: 250 °C				-	300

**Table 4:** EDS analysis result of Carbon and Tin.

Map Sum Spectrum				
Element	Line Type	Wt%	Wt% Sigma	Atomic %
C	K series	95.98	0.12	99.58
Sn	L series	4.02	0.12	0.42
Total:		100.00		100.00

## Data Availability

The original contributions presented in this study are included in the article. Further inquiries can be directed to the corresponding author.
